# Intranasal Delivery of a Chitosan-Hydrogel Vaccine Generates Nasal Tissue Resident Memory CD8^+^ T Cells That Are Protective against Influenza Virus Infection

**DOI:** 10.3390/vaccines8040572

**Published:** 2020-10-01

**Authors:** James G. Bedford, Irina Caminschi, Linda M. Wakim

**Affiliations:** 1 Department of Microbiology and Immunology, Peter Doherty Institute for Infection and Immunity, The University of Melbourne, Melbourne 3000, Australia; bedford.j@unimelb.edu.au (J.G.B.); irina.caminschi@monash.edu (I.C.); 2 Infection and Immunity Program, Monash Biomedicine Discovery Institute and Department of Biochemistry and Molecular Biology, Monash University, Clayton 3800, Australia

**Keywords:** resident memory T cells, influenza vaccines, intranasal vaccines

## Abstract

Rapid antigen clearance from the nasal mucosa is one of the major challenges in the development of intranasal vaccines. Here, we tested whether intranasal immunization with a chitosan-hydrogel vaccine, with in situ gelling properties, extended antigen retention time within the nasal mucosa. Intranasal immunization with a chitosan-hydrogel vaccine retained antigen within the upper respiratory tract (URT), while intranasal delivery of less viscous vaccines led to antigen accumulation within the lower airways. Interestingly, sustained antigen retention within the URT following chitosan-hydrogel vaccination boosted the number of vaccine-specific, tissue resident memory (Trm) CD8^+^ T cells that developed within the nasal mucosa. Mice immunized with a chitosan-hydrogel vaccine loaded with influenza virus peptides developed a large pool of influenza-specific CD8^+^ nasal Trm and these cells were highly protective during an influenza challenge. Our results describe an effective vaccine formulation that can be utilized to boost local immunity in the nasal mucosa.

## 1. Introduction

The intranasal delivery of vaccines offers many benefits over the traditional injectable regimes. Intranasal vaccination is cost effective, noninvasive, and does not require skilled professionals for administration. While nasal delivery is considered an attractive route for vaccine administration, the microenvironment of the upper respiratory tract presents a challenge, with various factors limiting antigen absorption and retention time in the nasal cavity. Most antigens have little/no affinity for the nasal epithelium and tend to be removed quickly from this region by mucociliary clearance. Extending the nasal residence time of the antigen by coadministering it with a viscous mucoadhesive is one approach to increase contact time between the antigen and the epithelial tissue in the nasal mucosa. Chitosan is a naturally occurring mucoadhesive polymer that is widely exploited for biomedical applications due to its good biocompatibility and low immunogenicity [1]. Chitosan-based in situ gelling systems are already a common biomaterial used in sustained drug delivery systems [2]. Chitosan solutions can be made thermosensitive with the addition of pluronics so that the hydrogels only form as a result of an increase in temperature [3]. This behavior can be exploited to produce chitosan solutions that are liquids at room temperature, enabling simple incorporation of vaccine antigens, but transform into gels when heated to body temperature.

In the present study, we tested whether intranasal immunization with a chitosan-hydrogel-based vaccine, with in situ gelling properties, extended antigen retention time within the nasal mucosa, and explored the impact this had on the development of local immunity.

## 2. Materials and Methods

### 2.1. Mice and Infections

C57BL/6 (CD45.2) and OT-I.CD45.1, mice were bred in-house and housed in specific pathogen-free conditions in the animal facility at the Doherty Institute of Infection and Immunity, the University of Melbourne. All experiments were done in accordance with the Institutional Animal Care and Use Committee guidelines of the University of Melbourne. Mice either received intranasally 10^4.5^ PFU of X31 (H3N2) in a volume of 10 μL to limit the virus to the upper respiratory tract or were infected via the intraperitoneal route with 10^6^ PFU of A/Puerto Rico/8 (H1N1).

### 2.2. Chitosan Vaccine Solution

Medium molecular weight (MW) chitosan (m.wt. 400,000), poloxamer 188 (Pluronic F68, m.wt: 8400) and poloxamer 407 (Pluronic F127, m.wt: 12,500) were purchased from Sigma (Sigma, Saint-Quentin, France). Chitosan (1% *w*/*v*) dissolved in 2% acetic acid solution was cooled to 4 °C. Poloxamer 407 and poloxamer 188 were added with continuous stirring to a 0.1% (*w*/*v*) solution of chitosan, so that the final concentrations of each polymer were 18% and 9–10% (*w*/*v*), respectively. The gelling temperature of the vaccine formulation was determined by placing the solution into a heat block and increasing the temperature at a rate of 1 °C per 5 min. The gel state was defined as no liquid flow if inverted. The amount of antigen loaded into the base vaccine formulation was the minimal amount of antigen we could incorporate into the solution without impacting viscosity, and that generated a highly reproducible response. Ovalbumin protein (Sigma) and lipopolysaccharide (LPS, Sigma) were added for a final concentration of 1.5 mg/mL and 50 μg/mL, respectively, and the solution was stirred at 4 °C to ensure uniform distribution. Mice were anaesthetized with inhalation anesthetic and immunized intranasally with the vaccine solution in a volume of 20 μL, which equated to 30 μg of antigen per dose. For influenza virus challenge experiments, influenza virus nucleoprotein peptide (NP_366–374_; *ASNENMETM*) was added to chitosan-poloxamer base solution at a final concentration of 154 μg/mL to which LPS (Sigma) was added for a final concentration of 50 μg/mL. Mice were immunized intranasally with the vaccine solution in a volume of 20 μL, which equated to 3 μg of antigen per dose.

### 2.3. In Vitro Activation of OT-I Cells

Naïve OT-I CD8^+^ T cells isolated from OT-I TCR transgenic mice were purified as described previously [4]. Mice were seeded with 1 × 10^6^ Carboxyfluorescein succinimidyl ester (CFSE)-labelled naïve OT-I cells or 5 × 10^6^ in vitro activated OT-I cells. OT-I T cells were activated in vitro with 10^−6^ M SIINFEKL peptide as described previously [4].

### 2.4. Study Design

All experiments were performed two to three times, with more than 3 mice per experimental group. To assess where the vaccine antigen deposits after intranasal immunization, mice were sacrificed at timepoints between one and four days post immunization. To assess the location of T cell priming post intranasal vaccination, mice were sacrificed at day 3 post immunization as this reflects a timepoint when significant T cell division occurs at the primary site of T cell activation but prior to the egress of the cells into the circulation. To assess the deposition of resident memory T cells post vaccination, mice were sacrified at day 30 post immunization as this reflects a timepoint when the effector T cells have contracted into a stable memory T cell pool.

### 2.5. Flow Cytometry and Cytometric Bead Array

Single-cell suspensions were prepared from spleens and lymph nodes (LNs) by mechanical disruption. Mice were perfused before the harvest of the lung tissue and nasal tissue, which were digested for one hour at 37 °C in collagenase type 3 (Worthington, 3 mg/mL) supplemented with DNAse I (Roche, 0.3 mg/mL). Cells were stained on ice with the appropriate mixture of monoclonal antibodies and washed with PBS with 2% bovine serum albumin. To measure levels of IFNγ production, single-cell suspensions generated from tissue as described above, were stimulated in vitro with 1 μM SIINFEKL peptide for one hour prior to the addition of Golgi Plug for an additional three hours. Intracellular IFNγ was stained using a BD Cytofix/Cytoperm kit. The conjugated monoclonal antibodies were obtained from BD Pharmingen or Biolegend: CD8 (53–6.7), CD45.1 (A20), CD103 (2E7), CD69 (H1.2F3), Ly6G (1A8), CD11b (M1/70), IFNγ (XMG1.2). H2-Db-NP_366_ tetramer was made in house.

Nasal lavage fluid and bronchial alveolar lavage fluid (BALf) from mice were tested for the presence of a panel of inflammatory cytokines using a LegendPlex Mouse antivirus response cytometric bead array (Biolegend) following the manufacturer’s instructions.

### 2.6. Influenza Virus Plaque Assay

Plaque assay for influenza virus quantitation was performed as described previously [5].

### 2.7. Histological Staining

Nasal tissue sections were generated as previously described [6]. Frozen sections were rehydrated and then stained in hematoxylin and eosin.

### 2.8. Detection of Serum Antibodies by ELISA

ELISA plates (Costar) were coated overnight at 4 °C with 10 μg/mL Ovalbumin (OVA). Unbound protein was washed away (PBS, 0.05% Tween 20). Serially diluted serum samples (PBS, 5% milk powder) were plated and incubated at 4 °C overnight. Bound anti-OVA IgG antibodies were detected using donkey anti-mouse IgG-HRP (Chemicon International) and visualized using ABTS as described previously [7]. Optical density was measured at 405–490 nm and endpoint titres were defined as the first dilution above background.

## 3. Results and Discussion

### 3.1. Intranasal Immunization with a Chitosan-Hydrogel Vaccine Retains Antigen in the Nasal Mucosa and Results in CD8^+^ T Cell Priming in the Lymph Nodes that Drain the Upper Respiratory Tract

**Figure 1:**
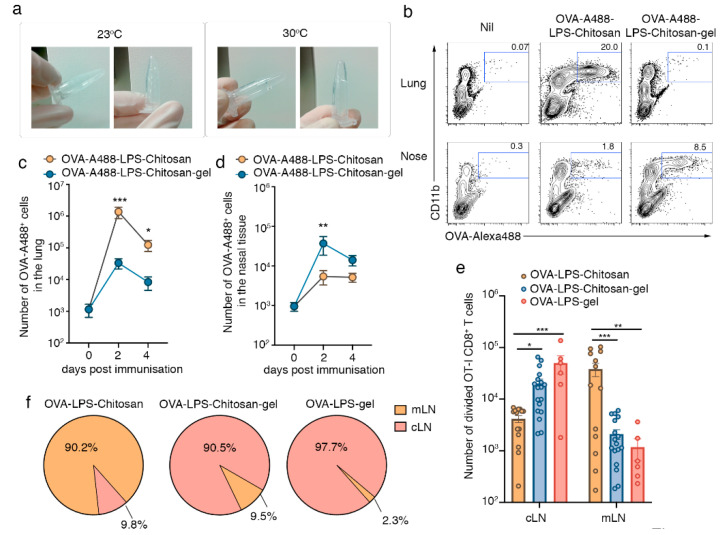
Intranasal immunization with a chitosan-hydrogel vaccine results in CD8^+^ T cell priming in lymph nodes draining the upper respiratory tract. (**a**) Chitosan hydrogel at room temperature and at 30 °C. (**b**–**d**) Lung and nasal tissue was recovered from mice two or four days post intranasal immunization with OVA-A88-LPS-chitosan or OVA-A488-LPS-chitosan gel; (**b**) representative flow cytometry profiles, gated on live cells, depicting the proportion of OVA-A488^+^ cells in the lung and nose on day 2 post immunization. The absolute number of A488^+^ cells in the (**c**) lung and (**d**) nasal tissue was measured. Data pooled from three independent experiments. Symbols represent the mean + standard error of mean (SEM) (*n* = 4–6 mice per group; two-way ANOVA Sidak’s multiple comparison). (**e**,**f**) Mice were injected with 10^6^ naive CFSE labelled CD45.1^+^CD8^+^ OT-I T cells and then intranasally immunized with OVA-LPS-chitosan, OVA-LPS-chitosan gel or OVA-LPS gel and, three days later, the (**e**) absolute number of divided OT-I cells in the cervical (cLN) and mediastinal (mLN) lymph node was quantitated. Data pooled from four independent experiments. Symbols represent individual mice (*n* = 6–18 mice per group; two way ANOVA, Sidak’s multiple comparison) (**f**) Pie graphs depict the proportion of total divided cells localized to the cLN or mLN following vaccination with the different formulations. * *p* < 0.5, ** *p* < 0.01, *** *p* < 0.001.

To identify where antigen accumulates following intranasal immunization with a chitosan-hydrogel vaccine, we loaded the model antigen ovalbumin (OVA) conjugated to the fluorophore alexa-488 (A488) and the adjuvant LPS, into either the chitosan-hydrogel base solution, which has sol-gel properties at temperatures >30 °C (Figure 1a) or a chitosan solution without the key gelling components (the pluronics). Two and four days post immunization, we looked for signs of local inflammation and for the presence of fluorescent antigen in the upper and lower respiratory tract. On day 2 post intranasal immunization, histological staining of nasal tissue sections revealed that mice immunized with either formulation showed evidence of a local inflammatory response, with the nasal cavity lumen of both cohorts containing cellular aggregates (Figure S1). Consistent with this observation, flow cytometric profiling of the cells present in the nasal lavage revealed significant neutrophil egress into the airways (Figure S2) and elevated levels of a panel of inflammatory cytokines in the nasal and bronchial alveolar lavage fluid of both groups of immunized mice (Figure S3). In mice immunized with the OVA-A488-LPS-chitosan vaccine which lacked gelling properties, the vast majority of antigen was found within the lung tissue (Figure 1b,c). In comparison, most OVA antigen appeared to be retained within the nasal tissue of the upper respiratory tract when incorporated into the chitosan-hydrogel vaccine (Figure 1b,d). Quantitation of the absolute number of OVA-A488^+^ cells along the respiratory tract two days post immunization revealed 41-fold more antigen-positive cells in the lung of mice vaccinated with the OVA-A488-LPS-chitosan formulation in comparison to mice given the chitosan-hydrogel vaccine. In contrast, assessment of the nasal tissue at this same timepoint showed 6.7-fold more antigen-positive cells in the animals receiving the chitosan-hydrogel vaccine compared to animals that received the formulation that could not form a gel. Flow cytometric assessment of lung and nasal tissue homogenates two days post administration showed that the majority of fluorescent antigen was distributed among Ly6G ^+^  neutrophils and CD64 ^+^  macrophages, although by day 4 post immunization, the fluorescent antigen was solely associated with macrophages (Figure S4).

Next, we determined whether the deposition of antigen at different sites along the respiratory tract altered the location for cytotoxic T lymphocyte (CTL) priming. To this end, congenically marked (CD45.1) CFSE-labelled OVA-specific naïve OT-I T-cell receptor (TCR) transgenic CD8^+^ T cells were adoptively transferred into C57BL/6 recipients (CD45.2), which were then immunized in the upper respiratory tract with the chitosan-hydrogel vaccine loaded with OVA protein and LPS (OVA-LPS-Chitosan-gel). As a comparison, and to identify the essential components of the base vaccine formulation we also immunized mice with OVA and LPS-loaded versions of the vaccine which contained chitosan but lacked the pluronics, and thus did not form the gel (OVA-LPS-chitosan), or a version that contained the gel-forming pluronics but lacked the mucoadhesive, chitosan (OVA-LPS-gel). The absolute number of divided OT-I T cells (CFSE^lo^) in cervical LNs (cLNs, draining the upper respiratory tract), and mediastinal LNs (mLNs, draining the lower respiratory tract) was determined three days post immunization (Figure 1e,f). Nasal-associated lymphoid tissue was not assessed as we have previously shown that naïve T cells do not traffic through this mucosal associated lymphoid tissue [6]. In mice that received vaccine formulations that could form gels following delivery into the upper airways, the activation of CD8^+^ T cells was largely confined to the cLNs, as this was where more than 90.5% of OT-I cells had undergone division (Figure 1e,f). In contrast, following immunization with OVA-LPS-chitosan, more than 90% of OT-I cells that had undergone division were found in the mLNs, which service the lower airways. In summary, the chitosan-gel vaccine resulted in antigen retention within the nasal mucosa and this promoted T cell priming in the LN that drained the upper respiratory tract.

### 3.2. Intranasal Immunization with a Chitosan-Hydrogel Vaccine Results in the Development of Nasal Trm

**Figure 2:**
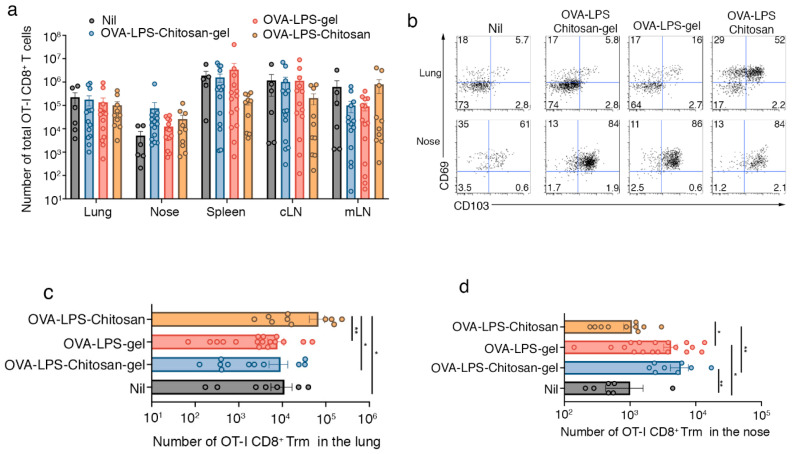
Intranasal immunization with a chitosan-hydrogel vaccine results in the development of nasal Trm. (**a**–**d**) Mice were seeded with 5 × 10^6^ in-vitro-activated CD45.1^+^CD8^+^OT-I T cells and then intranasally immunized with either saline (nil), OVA-LPS-chitosan, OVA-LPS-chitosan gel or OVA-LPS gel and, 30 days later, the (**a**) absolute number of total OT-I cells in the lung, nose, spleen, cLN and mLN was measured. (**b**) Representative flow cytometry profiles gated on OT-I.CD8^+^ T cells in the nose and lung show the expression levels of CD103 and CD69. (**c**,**d**) The absolute number of tissue resident (CD103^+^CD69^+^) OT-I in the (**c**) lung and (**d**) nose was determined. Data pooled from three independent experiments. Symbols represent individual mice, bars represent the mean + SEM (*n* = 7–18 mice per group; one-way ANOVA, Tukey’s multiple comparison). * *p* < 0.5, ** *p* < 0.01.

Next, we investigated whether the longer retention of antigen within the nasal mucosa following immunization with the hydrogel vaccines impacted the deposition of local T-cell immunity within the nasal tissue, specifically whether the regime boosted nasal CD8^+^ tissue resident memory (Trm) development. Trm are a self-sustaining tissue-bound population of memory T cells [8] that provide rapid protection against local secondary infections through direct effector functions [9,10] and by promoting the recruitment of circulating immune cells [11,12,13]. To this end, we seeded mice intravenously with in-vitro-activated effector OT-I cells and then boosted animals by intranasally delivering either OVA-LPS-chitosan gel, OVA-LPS gel, OVA-LPS-chitosan or, as a control, saline. We utilized this approach as it ensures all cohorts of mice had equal-sized populations of effector OT-I T cells, and therefore this would allow assessment of how the vaccine formulations would specifically impact respiratory tract Trm development. After 30 days, we enumerated the total number of OT-I, assessed the functionality of these cells, and measured the proportion that converted into Trm at various sites along the respiratory tract. While we did not observe any significant difference in the absolute number of memory OT-I CD8^+^ T cells in the spleen, cLN, mLN, nasal tissue and lung across all cohorts of mice (Figure 2a), or in the capacity of these cells to make the proinflammatory cytokine IFNγ following a brief in vitro stimulation (Figure S5), we did observe differences in Trm conversion (Figure 2b,c). The highest proportion (52%) and absolute number of lung OT-I Trm, defined by the coexpression of CD103 and CD69, developed following immunization with the OVA-LPS-chitosan vaccine. This made sense as local antigen presentation is required for pulmonary Trm development [14], and the OVA-LPS-chitosan intranasal vaccine was the only formulation that resulted in antigen accumulation within the lung tissue (Figure 2b,c). While nasal Trm can develop independently of in situ antigen recognition [5], the presence of cognate antigen at the site of development is known to boost Trm numbers [15]. Consistent with this, in all cohorts of mice, irrespective of whether they received an intranasal immunization, the vast majority (60–80%) of OT-I memory CD8^+^ T cells recovered from the nasal tissue were Trm (Figure 2b). While vaccination with OVA-LPS-loaded hydrogel vaccine formulations did not increase the proportion of nasal Trm above that observed following immunization with formulations that were not retained within the nasal tissue, they did boost the absolute number of Trm within this tissue by four- to fivefold (Figure 2b,d). Intranasal delivery of the hydrogel formulation that lacked the OVA antigen did not increase nasal Trm numbers (Figure S6). In addition to generating T cell immunity, all our vaccine formulations also generated equivalent levels of anti-OVA reactive antibodies, which we detected in the serum of vaccinated animals on day 14 post immunization (Figure S7).

This data indicates that altering the viscosity of intranasal vaccine formulation results in antigen retention at different locations along the respiratory tract, which, in turn, preferentially boosts Trm deposition at the site of antigen retention.

### 3.3. Intranasal Immunization with an Influenza Virus NP-peptide Loaded Chitosan Hydrogel Vaccine Results in the Development of Nasal Trm that are Protective against Influenza Virus Infection

**Figure 3:**
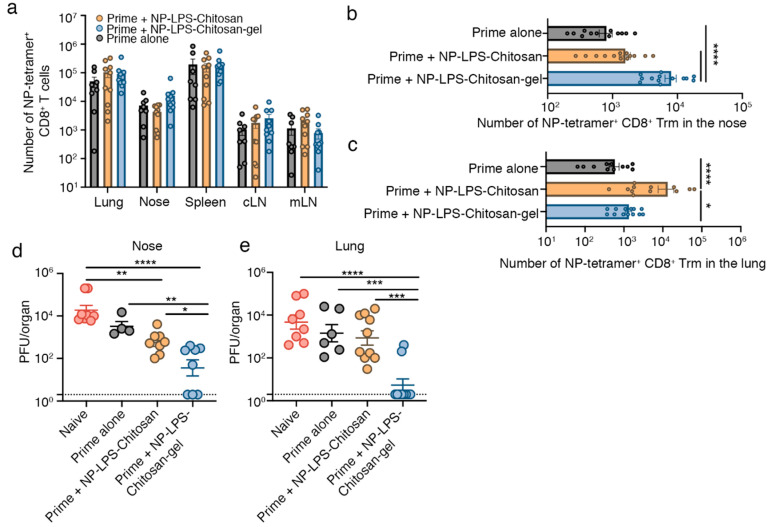
Intranasal immunization with an influenza virus nucleoprotein (NP)-peptide-loaded chitosan-hydrogel vaccine results in the development of nasal Trm that are protective against influenza virus infection. (**a**–**c**) Mice were primed by intraperitoneal injection of 10^6^ PFU of influenza A virus A/Puerto Rico/8 (H1N1) (PR8) strain of influenza virus, and 7 and 14 days later, boosted intranasally with either saline (Prime alone), NP-LPS-chitosan, or NP-LPS-chitosan gel. The absolute number of (**a**) total NP_366_-tetramer^+^ CD8^+^ T cells in the lung, nose, spleen, cLN and mLN and the absolute number of CD103^+^CD69^+^ Trm NP_366_-tetramer^+^ CD8^+^ T cells in the (**b**) nose and (**c**) lung was measured 30 days after the initial prime. Data pooled from three independent experiments. Symbols represent individual mice, bars represent the mean + SEM (*n* = 13–14 mice per group; one way ANOVA, Tukey’s multiple comparison) (**d**,**e**) Mice immunized as described above were challenged 28 days after the final boost with 10^4.5^ PFU of X31(H3N2) via an upper respiratory tract infection and, three days later, the viral titres in the (**d**) nasal tissue and (**e**) lung were determined. Data pooled from three independent experiments. Dots represent individual mice, lines represent the mean + SEM (*n* = 6–10 mice per group; one-way ANOVA, Dunnett’s multiple comparison). * *p* < 0.5, ** *p* < 0.01, *** *p* < 0.001, **** *p* < 0.0001.

Next, we investigated whether immunization with the chitosan-hydrogel vaccine would protect mice against a respiratory virus infection. To do this, we loaded the chitosan-hydrogel vaccine with influenza virus nucleoprotein (NP) peptide and adjuvant (LPS). Mice were firstly primed by intraperitoneal injection with the mouse-adapted influenza virus, A/Puerto Rico/8 (H1N1) (PR8), and then boosted 7 and 14 days later, by intranasal delivery of either NP-LPS-chitosan-gel, NP-LPS-chitosan, or as a control, saline. The absolute number of influenza NP-specific CD8^+^ T cells three weeks after the final boost were quantitated using H-2D^b^ tetramers loaded with the NP_366–374_ epitope. While we found no significant difference in the absolute number of NP_366_-tetramer^+^ CD8^+^ T cells in the lung, spleen, cLN, mLN and nasal tissue across all cohorts of mice (Figure 3a), once again, Trm development was influenced by the intranasal vaccine formulation. Mice primed and then boosted with the NP-LPS-chitosan gel vaccine had 10-fold more NP_366_-specific nasal Trm compared to mice that received the prime alone, or the NP-LPS-chitosan vaccine which was not efficiently retained within the upper airways (Figure 3b). By contrast, the largest population of influenza NP_366_-specific lung Trm developed following immunization with the NP-LPS-chitosan solution, which is consistent with this formulation largely resulting in antigen deposition within the lung (Figure 3c).

To test whether these vaccine formulations could provide protection against influenza virus infection, mice were immunized as described above, but this time were challenged in the upper respiratory tract 28 days after the final boost, with X31 (H3N2), a heterologous influenza A virus strain capable of migrating into the lower respiratory tract after upper respiratory tract infection [5]. At day 3 post infection (p.i.) with X31, mice were sacrificed, and viral titres in the respiratory tract were determined. Compared to naïve mice that received a primary infection with X31, mice receiving the prime alone (intraperitoneal PR8 cohort) did not display any significant reduction in viral titres in the nasal tissue or lung (Figure 3d,e), which is consistent with prior studies highlighting the importance of local Trm immunity in the control of influenza virus infection [16]. While we observed a 10-fold reduction in viral load in the nasal tissue of mice intranasally boosted with the NP-LPS-chitosan vaccine, we observed no reduction in the amount of influenza virus that migrated down into the lung tissue (Figure 3d,e). By contrast, mice immunized with the NP-LPS-chitosan-gel vaccine had significantly reduced X31 growth in the nasal tissue (100-fold reduction) and, in turn, 80% of mice had no detectable virus in the lung. This data highlights the effectiveness of strategies that lodge influenza specific Trm in the upper respiratory tract as an approach to limit the development of viral pneumonia in the lung.

For most respiratory pathogens, the nasal mucosa is the first barrier that must be crossed to enter the body. Despite the relevance of this site, it is an under investigated mucosal tissue and little is known of the immunity that can be evoked within this tissue or the immune mechanisms left behind that safeguard this region from respiratory pathogens. We have previously shown Trm persist within the nasal tissue following influenza A virus infection and these cells are highly effective at reducing the viral load in the upper airways [5]. Reducing the nasopharyngeal viral load has two benefits: it decreases viral transmission across a population by making individuals less contagious [17], and it can limit the spill-over of the virus into the lower respiratory tract, a scenario that often leads to more severe disease outcomes [5]. Strategies that lodge Trm in the nasal mucosa will limit the spread of respiratory pathogens, both across the population, and between the upper and lower airways.

Here we show that vaccines with increased viscosity have longer retention times in the nasal cavity, which, interestingly, in addition to directing CTL priming to the LN draining the upper airways, also boosted the development of local nasal Trm. Mice primed with an intraperitoneal injection and then boosted with an intranasal chitosan-hydrogel vaccine loaded with influenza virus peptides developed a large pool of influenza-specific CD8^+^ nasal Trm and these cells, post influenza challenge, significantly attenuated virus replication in the upper airways and blocked the development of viral pneumonia. While the vaccination regime presented here involved priming mice using an injectable vaccine, in future it will be important to determine whether alternative strategies whereby multiple intranasal vaccinations, or potentially a single intranasal vaccination with a formulation with extended longevity and antigen delivery into the upper airways, can also generate protective nasal Trm.

In this study, we loaded our intranasal vaccine formulations with the adjuvant LPS, which we acknowledge is unsuitable for clinical use. There are several licensed adjuvants that can promote effective immunity when used as part of an injectable vaccine, however there is a paucity of adjuvants that are effective and can be safely administered by the intranasal route. While this study largely focused on assessing whether viscosity and antigen retention within the nasal mucosae impacted the local immune response, moving forward, it will be important to identify an adjuvant that is safe for intranasal delivery or investigate whether the chitosan component of our formulation, which has adjuvant properties [18] can evoke comparable levels of immunity.

## 4. Conclusions

Overall, our results describe an effective vaccination strategy for depositing highly protective local immunity within the nasal mucosa and highlights the value of approaches that boost immunity in the upper airways for blocking the development of more severe lower respiratory tract infections. While we show here the chitosan-hydrogel-based vaccine is very effective at providing protection against influenza virus infection, this formulation could easily be adapted to evoke nasal CD8^+^ Trm that are reactive towards other clinically relevant respiratory viruses, including SARS-CoV-2.

## 5. Patents

L.W. is named as an inventor on a provisional patent application filed by the University of Melbourne covering the use of chitosan hydrogel as part of vaccine formulation against influenza virus (2020902546).

## Supplementary Materials

The following are available online at https://www.mdpi.com/2076-393X/8/4/572/s1, Figure S1: Mice were intranasally immunized with either saline (naive), OVA-LPS-chitosan, or OVA-LPS-Chitosan gel and, two days later, the nasal tissue sections were stained with hematoxylin and eosin (10x). Data representative of two independent experiments (*n* = 2); Figure S2: Mice were intranasally immunized with either saline (nil), OVA-LPS-chitosan, or OVA-LPS-chitosan gel and, two days later, the absolute number of neutrophils (Ly6G^+^CD11b^+^) cells in the nasal lavage was assessed. (a) Representative flow cytometry profiles showing neutrophils in the nasal lavage. (b) The absolute number of neutrophils in the nasal lavage. Symbols represent individual mice and bars represent means ± SEM (*n* = 4–5 mice per timepoint; one-way ANOVA, Tukey’s multiple comparison); Figure S3: Mice were intranasally immunized with either saline (nil), OVA-LPS-chitosan, or OVA-LPS-chitosan gel and, two days later, the level of a panel of cytokine/chemokines in the (a) nasal lavage fluid and (b) bronchial alveolar lavage fluid was measured by cytometric bead array. Bars represent means ± SEM and the dotted line represents the limit of detection (*n* = 3–5 mice per timepoint; two-way ANOVA, Tukey’s multiple comparison); Figure S4: Lung and nasal tissue were recovered from mice two or four days post intranasally immunization with OVA-A88-LPS-chitosan or OVA-A488-LPS-chitosan gel. Representative flow cytometry profiles, gated on live cells, OVA-A488+, CD11b+ cells in the lung and nose on day 2 and 4 post immunization; Figure S5: Mice were seeded with 5 × 10^6^ in-vitro-activated CD45.1+CD8+OT-I T cells and then intranasally immunized with either saline (nil), OVA-LPS-chitosan, or OVA-LPS-chitosan gel and, 30 days later, the lung, nose and spleen were harvested and the proportion of OT-I cells synthesizing IFNγ following a brief in vitro stimulation was assessed. Flow cytometry profiles, representative of two independent experiments, are shown; Figure S6: Mice were seeded with 5 × 10^6^ in-vitro-activated CD45.1+CD8+ OT-I T cells and then intranasally immunized with either saline (nil), OVA-LPS-chitosan, OVA-LPS-chitosan gel or LPS gel and, 30 days later, the absolute number of tissue resident (CD103+CD69+) OT-I in the lung and nose was determined. Data pooled from two independent experiments. Bars represent the mean + SEM (*n* = 3–8 mice per group; two-way ANOVA, Sidak’s multiple comparison); Figure S7: Mice were intranasally immunized with either saline (naive), OVA-LPS-chitosan, or OVA-LPS-chitosan gel and, 14 days later, serum anti-OVA Ig reactivity was measured by ELISA and end-point titres are depicted. The cumulative data of two independent experiments are depicted. Symbols represent individual mice and bars represent means ± SEM and the dotted line shows the limit of detection (*n* = 3–5 mice per group).
