# Supplementary material for: Intranasal Delivery of a Chitosan-Hydrogel Vaccine Generates Nasal Tissue Resident Memory CD8+ T Cells That Are Protective against Influenza Virus Infection
Source: Vaccines (Basel). 2020 Oct 1;8(4):572. doi: 10.3390/vaccines8040572 (PMC7712318; doi:10.3390/vaccines8040572)
Supplement: Supplementary file 1 [file vaccines-08-00572-s001.pdf]

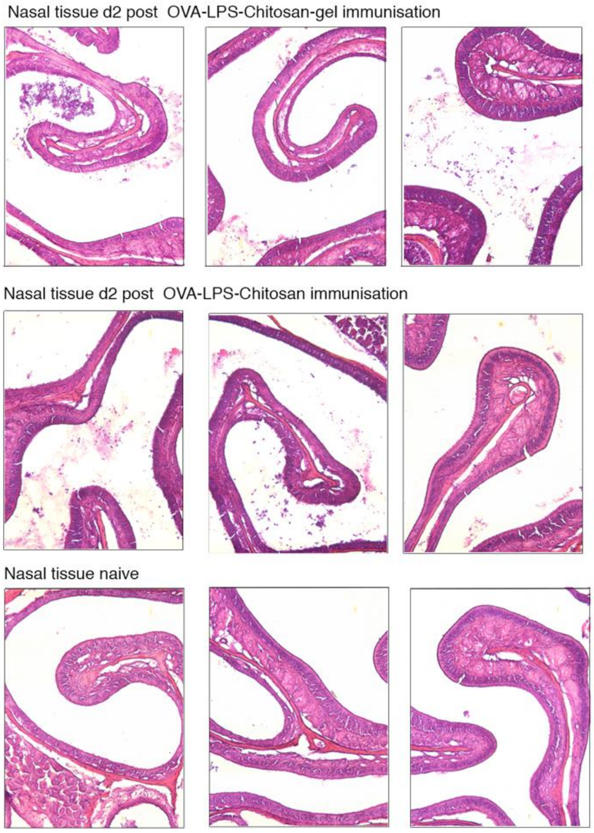

Supplementary Figure 1: Mice were intranasally immunized with either saline (naive), OVA-LPS-chitosan, or OVA-LPS-Chitosan gel and, two days later, nasal tissue sections were stained with hematoxylin and eosin (10x). Data representative of two independent experiments (*n* = 2).

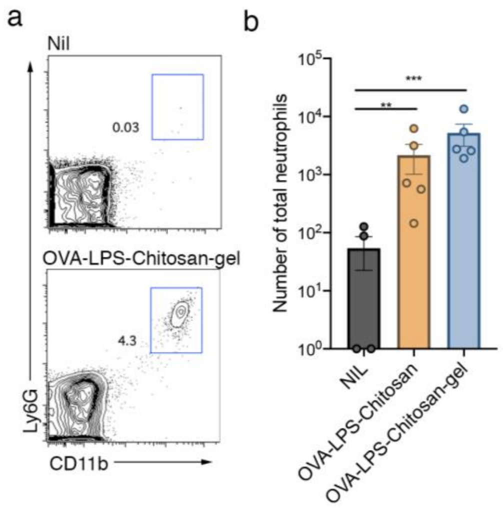

Supplementary Figure 2. Mice were intranasally immunized with either saline (nil), OVA-LPS-chitosan, or OVA-LPS-chitosan gel and, two days later, the absolute number of neutrophils (Ly6G^{+}CD11b^{+}) in the nasal lavage was assessed. (a) Representative flow cytometry profiles showing neutrophils in the nasal lavage. (b) The absolute number of neutrophils in the nasal lavage. Symbols represent individual mice and bars represent means ± SEM (*n* = 4–5 mice per timepoint; one-way ANOVA, Tukey's multiple comparison).

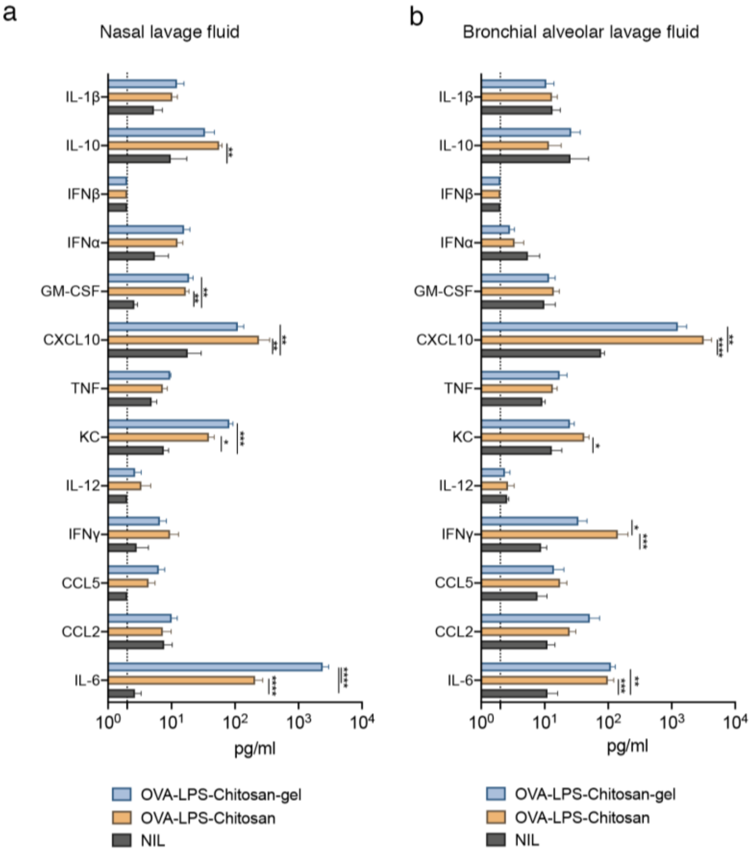

Supplementary Figure 3: Mice were intranasally immunized with either saline (nil), OVA-LPS-chitosan, or OVA-LPS-chitosan gel and, two days later, the level of a panel of cytokine/chemokines in the (a) nasal lavage fluid and (b) bronchial alveolar lavage fluid was measured by cytometric bead array. Bars represent means ± SEM and the dotted line represents the limit of detection (n = 3–5 mice per timepoint; two-way ANOVA, Tukey's multiple comparison).

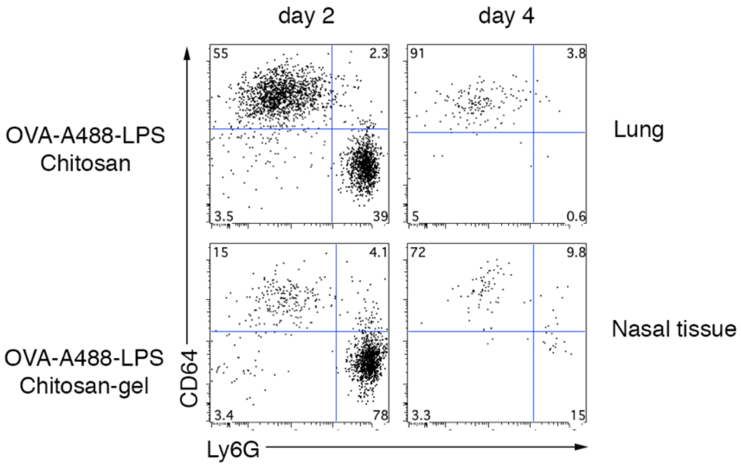

Supplementary Figure 4: Lung and nasal tissue were recovered from mice two or four days post intranasal immunisation with OVA-A88-LPS-chitosan or OVA-A488-LPS-chitosan gel. Representative flow cytometry profiles, gated on OVA-A488$^+$CD11b$^+$ cells in the lung and nose on day 2 and 4 post immunization.

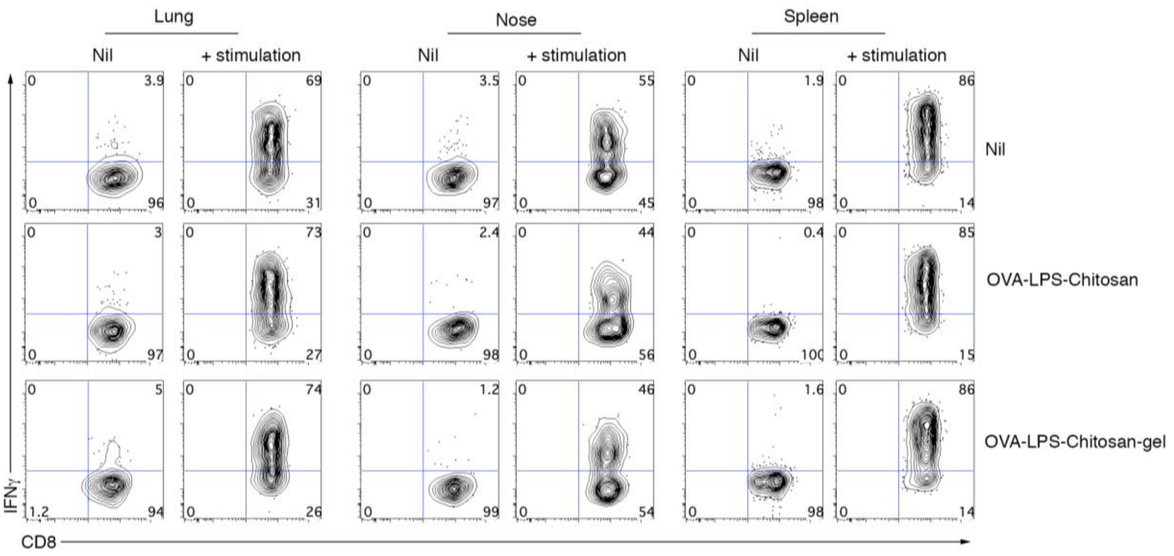

Supplementary Figure 5. Mice were seeded with 5 × 10^6 in-vitro-activated CD45.1$^+$CD8$^+$OT-I T cells and then intranasally immunized with either saline (nil), OVA-LPS-chitosan, or OVA-LPS-chitosan gel and, 30 days later, the lung, nose and spleen were harvested and the proportion of OT-I cells synthesizing IFNγ following a brief in vitro stimulation was assessed. Flow cytometry profiles, representative of two independent experiments, are shown.

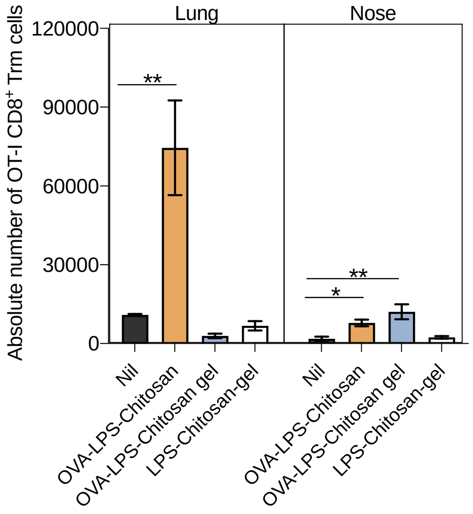

Supplementary Figure 6. Mice were seeded with 5 × 10^6 in-vitro-activated CD45.1$^+$CD8$^+$ OT-I T cells and then intranasally immunized with either saline (nil), OVA-LPS-chitosan, OVA-LPS-chitosan gel or LPS gel and, 30 days later, the absolute number of tissue resident (CD103$^+$CD69$^+$) OT-I in the lung and nose was determined. Data pooled from two independent experiments. Bars represent the mean + SEM (n = 3–8 mice per group; two-way ANOVA, Sidak's multiple comparison).

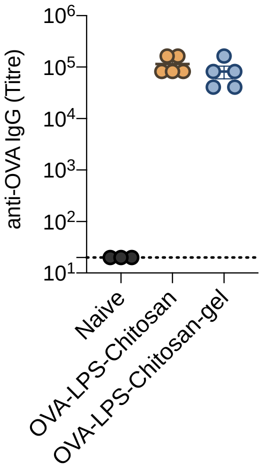

Supplementary Figure 7. Mice were intranasally immunized with either saline (naive), OVA-LPS-chitosan, or OVA-LPS-chitosan gel and, 14 days later, serum anti-OVA Ig reactivity was measured by ELISA and end-point titres are depicted. The cumulative data of two independent experiments are depicted. Symbols represent individual mice and bars represent means ± SEM and the dotted line shows the limit of detection (n = 3–5 mice per group).
